# LapTrack: linear assignment particle tracking with tunable metrics

**DOI:** 10.1093/bioinformatics/btac799

**Published:** 2022-12-10

**Authors:** Yohsuke T Fukai, Kyogo Kawaguchi

**Affiliations:** Nonequilibrium Physics of Living Matter RIKEN Hakubi Research Team, RIKEN Center for Biosystems Dynamics Research, Kobe 650-0047, Japan; Nonequilibrium Physics of Living Matter RIKEN Hakubi Research Team, RIKEN Center for Biosystems Dynamics Research, Kobe 650-0047, Japan; RIKEN Cluster for Pioneering Research, Kobe 650-0047, Japan; Universal Biology Institute, The University of Tokyo, Tokyo 113-0033, Japan

## Abstract

**Motivation:**

Particle tracking is an important step of analysis in a variety of scientific fields and is particularly indispensable for the construction of cellular lineages from live images. Although various supervised machine learning methods have been developed for cell tracking, the diversity of the data still necessitates heuristic methods that require parameter estimations from small amounts of data. For this, solving tracking as a linear assignment problem (LAP) has been widely applied and demonstrated to be efficient. However, there has been no implementation that allows custom connection costs, parallel parameter tuning with ground truth annotations, and the functionality to preserve ground truth connections, limiting the application to datasets with partial annotations.

**Results:**

We developed LapTrack, a LAP-based tracker which allows including arbitrary cost functions and inputs, parallel parameter tuning and ground-truth track preservation. Analysis of real and artificial datasets demonstrates the advantage of custom metric functions for tracking score improvement from distance-only cases. The tracker can be easily combined with other Python-based tools for particle detection, segmentation and visualization.

**Availability and implementation:**

LapTrack is available as a Python package on PyPi, and the notebook examples are shared at https://github.com/yfukai/laptrack. The data and code for this publication are hosted at https://github.com/NoneqPhysLivingMatterLab/laptrack-optimisation.

**Supplementary information:**

Supplementary data are available at *Bioinformatics* online.

## 1 Introduction

Automated tracking of particles in timelapse images is important in a wide range of fields in science and is especially crucial in creating large datasets of cell lineages in biological studies. Recently there has been considerable development in tracking algorithms, where methods based on probabilistic modeling (Bise *et al.*, 2011; Bove *et al.*, 2017; Chen, 2021; Chenouard *et al.*, 2009, 2014; Meijering *et al.*, 2009; Ulicna *et al.*, 2021; Ulman *et al.*, 2017) and supervised machine learning (Ben-Haim and Riklin-Raviv, 2022; Chen, 2021; Lou and Hamprecht, 2011; Ulman *et al.*, 2017) are increasingly being developed. The diverse nature of live imaging tasks, however, frequently requires tracking without underlining model or large-scale ground-truth annotations, emphasizing the need for a robust tracking algorithm with a small number of parameters that can be tuned by manual annotations.

Defining and optimizing a global cost function to appropriately penalize wrong connections is a common approach in robust tracking methods. If the cost function is a linear sum of the costs associated with the connections, we can employ efficient algorithms (Jonker and Volgenant, 1987; Kuhn, 1955) to solve the global optimization problem called the linear assignment problem (LAP). The LAP-based tracking method has proven to be accurate and robust, especially for data with higher particle density. To deal with particle splitting (by division or oversegmentation) or merging (by undersegmentation), which is common in live cell data, Jaqaman *et al.* (2008) further developed a two-stage LAP method, with the second stage dedicated to the connection of splitting and merging branches. The cost function in their case was the squared Euclidean distance between the positions of the objects, with additional intensity-associated costs for splitting and merging.

Tools have been developed to provide similar LAP-based algorithms with splitting and merging detection; TrackMate (Ershov *et al.*, 2022; Tinevez *et al.*, 2017), for example, provides distance-based LAP tracker with particle detection and segmentation workflow and a method to conduct manual correction, all within the Java-based framework in ImageJ (Schindelin *et al.*, 2012; Schneider *et al.*, 2012). Cell-ACDC (Padovani *et al.*, 2022), which was originally designed for yeast analysis, also implements an overlap-based LAP tracker with splitting detection, as well as various functions ranging from image alignment to manual correction that support the entire analysis workflow in Python. In addition, TracX (Cuny *et al.*, 2022) employs a multi-round tracking and correction workflow using a LAP tracker and mistracking detector by matching image features.

Although other highly accurate methods have been proposed to work for the tracking problem with cell divisions, no single tracking algorithm will be perfect for all the diverse experimental situations (Ulman *et al.*, 2017). To obtain near-perfect segmentation and tracking for specific data, users must still optimize the segmentation and tracking steps, automatically or manually. In this regard, the LAP-based algorithm that robustly works with a small number of parameters continues to play a key role in generating the initial tracking data without large-scale manual annotation.

An adaptive improvement to the original LAP-based tracking with distance can be made by using additional features taken from the cell images. For example, we can extract the morphology of each cell, such as its shape and size, from typical live cell images, as well as the signal levels from multiple fluorescent channels. The consistency of cell shape and fluorescent signals across time frames is useful when tracking is conducted by human eyes, especially when the frame rate of the data is not high enough. Therefore, it is desirable to be able to implement arbitrary inputs and cost functions in the LAP-based tracking scheme, as well as to tune the parameters using partial ground-truth annotations.

These requirements motivated us to build a tool that recapitulates the LAP algorithm (Jaqaman *et al.*, 2008; Tinevez *et al.*, 2017) with additional flexibility and modularity; LapTrack is designed as a simple intermediate in the entire tracking pipeline that takes the positions and features of particles and returns LAP-optimized tracks. The three unique features of LapTrack are (i) arbitrary tunable cost functions for particle connection, (ii) integrability with other Python tools and (iii) the functionality to preserve ground-truth (annotated) connections. Within this framework, we can implement user-defined cost functions for connections that can take an arbitrary number of inputs. The tracking function is modularized and documented as an application programming interface (API) so that it can be integrated into any custom workflow in Python, allowing parallel parameter optimization as well as visualization of results in easy steps.

In this article, we demonstrate how this pipeline can be used not only to optimize the tracking in a supervised manner, but how it is also useful for efficient manual correction of the tracks when combined with visualization tools such as napari (Sofroniew *et al.*, 2022).

## 2 Materials and methods

### 2.1 Datasets

We here describe the data that we used to demonstrate the use cases of LapTrack: live cell images with ground truth segmentation and tracking (mouse paw epidermis dataset, cell migration dataset, Yeast Image Toolkit dataset and C2C12 dataset) and simulated data (colored particles) provided in https://github.com/NoneqPhysLivingMatterLab/laptrack-optimisation. We also used high-density vesicles, yeast and 3D *Drosophilla* data to show that the tracking pipeline works for a wide range of applications.

#### 2.1.1 Mouse paw epidermis dataset

The segmentation data and the ground truth tracking result collected and analyzed in Mesa *et al.* (2018); Yamamoto *et al.* (2022) were used as a reference. The dataset contains 236 to 327 cells in the observation area and has 15 frames.

#### 2.1.2 Cell migration dataset

Images, segmentation data for a portion of frames and the ground truth tracking result were downloaded from Zenodo (Pylvänäinen *et al.*, 2022). Segmentation was conducted by Cellpose (Stringer *et al.*, 2021) and manually corrected in napari (Sofroniew *et al.*, 2022). The ground-truth tracking result was also manually validated and corrected. The dataset contains 218 to 434 cells in the 648.95 µm × 648.95 µm observation area and has 86 frames.

#### 2.1.3 Yeast image toolkit dataset

The dataset was downloaded from the Yeast Image Toolkit website http://yeast-image-toolkit.org/ (Versari *et al.*, 2017). The data included the ground-truth cell positions at each time frame, which were used for the tracking in the benchmark (Section 3.2).

#### 2.1.4 C2C12 dataset

The dataset (Ker *et al.*, 2018) was downloaded from the public repository (Ker, 2017). We used the first 780 frames of sequence 9 with the ‘BMP2’ condition for the benchmark (Section 3.2), since it included the annotation for all cells in the field. We manually validated the dataset and removed duplicated annotations on a single cell.

#### 2.1.5 Colored particles

We simulated the Brownian motion of 400 particles with colors in a 2D box of size 20 × 20 with periodic boundary conditions. The particles were split into two species, *a* and *b*, where the interaction between the particles was set as harmonic repulsion with the spring constants set as 1 for *a* and *a* pairs, 1.2 for *a* and *b* pairs, and 1.4 for *b* and *b* pairs. The dynamics was simulated with the simulate.brownian routine in Jax-MD (Schoenholz and Cubuk, 2020) with the parameters *kT *=* *0.1 and *dt *=* *0.001, where the mass and friction coefficient were set to the default values, 1 and 0.1. For each particle, labeled by *i*, a random integer *n_i_* between 0 and 7 is assigned. The *feature vector* $c_{i}\in R^{3}$, corresponding to RGB colors, of each particle at each time step is then assigned as $c_{i}=\left(R\left(n_{i}^{3}\right),R\left(n_{i}^{2}\right),R\left(n_{i}^{1}\right)\right)$, where $n_{i}^{k}$ is the *k*th digit of *n_i_* in the binary representation and $R(x)=\delta_{x,0}N\left(2,0.5\right)+\delta_{x,1}N\left(6,0.5\right)$, where N(μ,σ) is the normal random variable with mean *μ* and the standard deviation *σ*. When used for the tracking benchmark, particles crossing the boundary are regarded as disconnected and belong to different tracks.

#### 2.1.6 Demonstration

The simulated single-molecule dataset was downloaded from the Particle Tracking Challenge website http://bioimageanalysis.org/track/ (Chenouard *et al.*, 2014). We used the high-density vesicles dataset with the signal-to-noise ratio* *7. Blobs were detected by the Laplacian-of-Gaussian (LoG) detector, skimage.feature.blob_log function in scikit-image (van der Walt *et al.*, 2014), with the parameters min_sigma = 1, max_sigma = 5, num_sigma = 5 and threshold = 0.05. The detected points were tracked by LapTrack with track_cost_cutoff = 100.

**Fig. 1.**
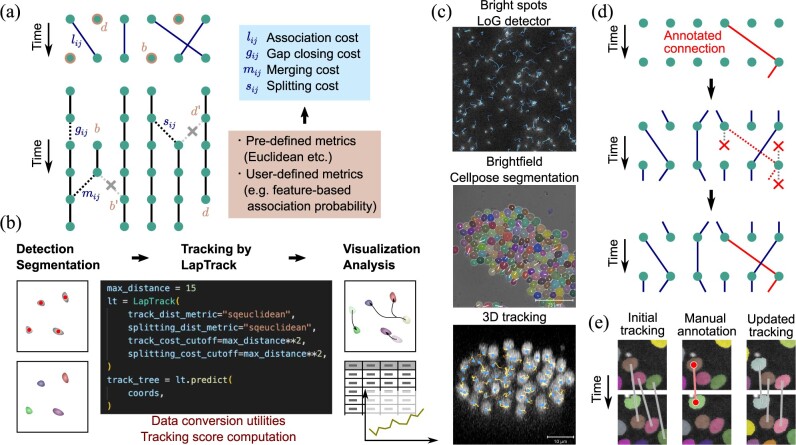
(**a**) The schematic for the tracking algorithm (see main text). (**b**) Expected workflow for cell segmentation, tracking and analysis using tools in Python. The particle detection or segmentation results can be directly supplied to LapTrack. The tracking result can be directly visualized and analyzed in Python. (**c**) Examples of tracks generated by LapTrack. The lines indicate the result tracks. (Top) The dataset from Particle Tracking Challenge, detected by the LoG detector. (Middle) The dataset from the Yeast Image Toolkit website, detected by Cellpose. (Bottom) The *Caenorhabditis elegans* developing embryo dataset from the Cell Tracking Challenge website. (**d**) The schematic for the tracking algorithm with freezing annotated connections. (Top) Annotated connections (red solid lines). (Middle) Connections from (to) a point that has an annotated connection from (to) itself are forbidden. (Bottom) The verified connections are added to the tracking tree. The split and merges are treated similarly. (**e**) Illustration of the manual-correction-aware tracking with napari (see main text) using the cell migration dataset. (Left) Original tracking result with mistakes (gray solid lines). (Middle) Annotation points are added in napari (red points) to specify a correct connection (red solid line). (Right) Updated tracking result after annotation. The annotated track as well as tracks nearby are automatically corrected (gray solid lines) (A color version of this figure appears in the online version of this article)

For Figure 1c, the Yeast Image Toolkit data in IT-Benchmark2/TestSet4/RawData were segmented by Cellpose 0.7.2 (Stringer *et al.*, 2021) with the parameters model_type=‘cyto’, net_avg=True, and diameter = 30 in the eval function. The centroids of each segmented region were tracked by LapTrack with the default metric and track_cost_cutoff = 100, splitting_cost_cutoff = 2500.

The 3D *Drosophilla* dataset (Fluo-N3DH-CE) was downloaded from the Cell Tracking Challenge website http://celltrackingchallenge.net/ (Ulman *et al.*, 2017). The data included marked cell positions in each time frame, which were connected to generate tracks by LapTrack with track_cost_cutoff = 10000, splitting_cost _cutoff = 2500.

### 2.2 Tracking implementation

The implemented particle tracking algorithm follows the method proposed in Jaqaman *et al.* (2008), with modifications following TrackMate (Ershov *et al.*, 2022; Tinevez *et al.*, 2017) and additional flexibility, as we describe in the following sections.

#### 2.2.1 Frame-to-frame LAP

In the first step, the points in successive frames are connected by solving LAP, and then generating tracks without splits and merges (Fig. 1a, left top). Specifically, for every pair of points with properties (such as Euclidean coordinates) *x_i_* and *x_j_* at frames *t* and *t *+* *1, the costs $l_{ij}=l(x_{i},x_{j})$ are computed using a user-definable metric function *l*. The costs *d* and *b* are then assigned to the particles not connected to any of the particles in the next and previous timesteps, respectively. The optimal assignment is found by minimizing the cost (Jaqaman *et al.*, 2008):

(1) $$L_{\text{ff}}=\sum_{(i,j)\in C}\left(l_{ij}+l_{0}\right)+Dd+Bb,$$

where C is the set of all connected index pairs, *B* and *D* are the numbers of the points which does not have a connection to the previous and next timesteps, respectively, and $l_{0}=\min\left(l_{ij},d,b\right)$ (see Supplementary Material for algorithm details). In the default setting, *d* and *b* are calculated as $1.05\times c^{90\%}$, where $c^{90\%}$ is the 90% percentile value of the all finite entries in ${\left\{l_{ij}\right\}}_{ij}$ (Jaqaman *et al.*, 2008). The default metric for *l* is set to the squared Euclidean distance $l(x_{i},x_{j})={\left\|x_{i}-x_{j}\right\|}_{2}^{2}$ (Ershov *et al.*, 2022; Jaqaman *et al.*, 2008; Tinevez *et al.*, 2017) with which the cost-minimizing association can be interpreted as the maximum log-likelihood solution for Brownian particles when we ignore splitting and merging (Crocker and Grier, 1996).

#### 2.2.2 Segment-connecting LAP

In the second step, another LAP is solved to predict splitting, merging, and gap closing (Fig. 1a, left bottom). Gap closing connects free segment ends with allowing frame skips. The gap closing cost $g_{\alpha \beta }=g(x_{\alpha },x_{\beta })$ is calculated by a user-definable metric *g* for all possible connections between free ends up to a specified frame difference, and the splitting and merging costs $s_{\alpha \beta }=s(x_{\alpha },x_{\beta })$ and $m_{\alpha \beta }=m(x_{\alpha },x_{\beta })$ are calculated for all possible connections between a free end and a track midpoint by user-definable metrics *s* and *m*. The metrics *g*, *s* and *m* default to the squared Euclidean distance. Then, the optimal assignment is calculated by minimizing the overall cost:

(2) $$\begin{array}{c} L_{\text{sc}}=\sum_{(\alpha ,\beta )\in G}\left(g_{\alpha \beta }+l_{0}^{\prime }\right)+\sum_{(\alpha ,\beta )\in S}\left(s_{\alpha \beta }+l_{0}^{\prime }\right)+\sum_{(\alpha ,\beta )\in M}\left(m_{\alpha \beta }+l_{0}^{\prime }\right)\\ +Dd+Bb+D\prime d\prime +B\prime b\prime , \end{array}$$

where G, S and M are the set of all gap-closing, splitting and merging index pairs, *D* and *B* are the number of the unconnected track ends and starts, D′ and B′ are the number of the track middle points that are not connected to other track ends as the split and merge, respectively (costs d′ and b′ are assigned to them, respectively) and $l_{0}^{\prime }=\min\left(g_{\alpha \beta },s_{\alpha \beta },m_{\alpha \beta },d,b,d\prime ,b\prime \right)$ (see Supplementary Material for details). In the default setting, *d*, *b*, d′ and b′ are calculated analogously to the frame-to-frame LAP.

#### 2.2.3 Freezing annotated tracks

We implemented an option to specify partial tracks within the data to be fixed as ground-truth verified connections (Fig. 1d). Fixing the correct tracks is especially useful when performing manual corrections using visualization tools such as napari. As we demonstrate (https://github.com/NoneqPhysLivingMatterLab/laptrack-optimisation) (Fig. 1e), track connections can be specified to be fixed by annotating the cell regions before rerunning the LAP-based tracking. The resulting track preserves the training data tracks due to the masking scheme (Fig. 1d).

#### 2.2.4 Parameter optimization

In practice, we introduce the cut-off for the costs *l_ij_*, *g_αβ_*, *s_αβ_* and *m_αβ_*, above which those values are regarded as infinity. The values of the cut-offs can affect the performance as demonstrated in Section 3.1, but it is difficult to optimize those values due to the non-differentiability of the LAP algorithm (Xu *et al.*, 2020) and the high computational cost for repeating the tracking routine. We therefore used non-gradient optimization methods to optimize the specified sets of the parameters in parallel using the package Ray Tune (Moritz *et al.*, 2018) with the Optuna optimizer (Akiba *et al.*, 2019) and random search. We selected the parameters that achieved the highest connection Jaccard index value or true positive rate, depending on the type of the training data (Section 2.3.1).

#### 2.2.5 Analysis pipeline

LapTrack is written in Python with explicit API documentation and can be integrated with, for example, particle detectors in scikit-image and deep learning-based segmentation packages such as Cellpose (Stringer *et al.*, 2021) (Fig. 1b and c). The output data is a networkx (Hagberg *et al.*, 2008) directed graph, which can be analyzed using the network analysis functions in the package. We also implemented utilities to convert data into pandas dataframes (pandas development team, 2020; Wes McKinney, 2010) and shorthand functions to track coordinates organized in a dataframe. In this paper, we used the ground-truth segmentation for each dataset as the input and analyzed the result tracks by networkx and pandas. Python scripts for tracking and analysis are provided at https://github.com/NoneqPhysLivingMatterLab/laptrack-optimisation.

### 2.3 Metrics for the tracking results

To measure the performance of tracking, we employed the following metrics, which can also be calculated within LapTrack.

#### 2.3.1 Overall tracking scores

To measure the overall track consistency, we calculated the *target effectiveness* (TE) and *track purity* (TP) (Bise *et al.*, 2011; Chen, 2021), which penalize the false negative and the false positive detections, respectively. Let us denote the set of ground truth tracks by ${\left\{T_{j}^{g}\right\}}_{j}$ and predicted tracks by ${\left\{T_{j}^{p}\right\}}_{j}$. TE for a single ground truth track $T_{j}^{g}$ is calculated by finding the predicted track $T_{k}^{p}$ that overlaps with $T_{j}^{g}$ in the largest number of the frames and then dividing the overlap frame counts by the total frame counts for $T_{j}^{g}$. The TE for the total dataset is calculated as the mean of TEs for all ground truth tracks, weighted by the length of the tracks. TP is defined analogously, with $T_{j}^{g}$ and $T_{j}^{p}$ being swapped in the definition. We also measured the *mitotic branching correctness* (Bise *et al.*, 2011; Chen, 2021), defined as the fraction of the number of correctly detected divisions over the total number of the divisions.

#### 2.3.2 Overlap between predicted and ground truth connections

During the parameter optimization, we used a less computationally expensive quantity, the *Jaccard index* and the *true positive rate* of the connections to measure how well the predicted connections overlap with the ground truth. The quantity is defined by $\left|E^{p}\cap E^{g}\right|/\left|E^{p}\cup E^{g}\right|$ and $\left|E^{p}\cap E^{g}\right|/\left|E^{g}\right|$, respectively, where we denote the set of predicted and ground-truth connections by $E^{p}$ and $E^{g}$, respectively, and the size of a set E by |E|. In the benchmark of the Yeast Image Toolkit dataset (Section 3.2), we additionally calculated the F-score of the assignment $2\left|E^{p}\cap E^{g}\right|/\left(\left|E^{p}\right|+\left|E^{g}\right|\right)$ to compare the performance with previously reported results.

## 3 Results

### 3.1 Distance cut-off points can be optimized to increase performance

**Fig. 2.**
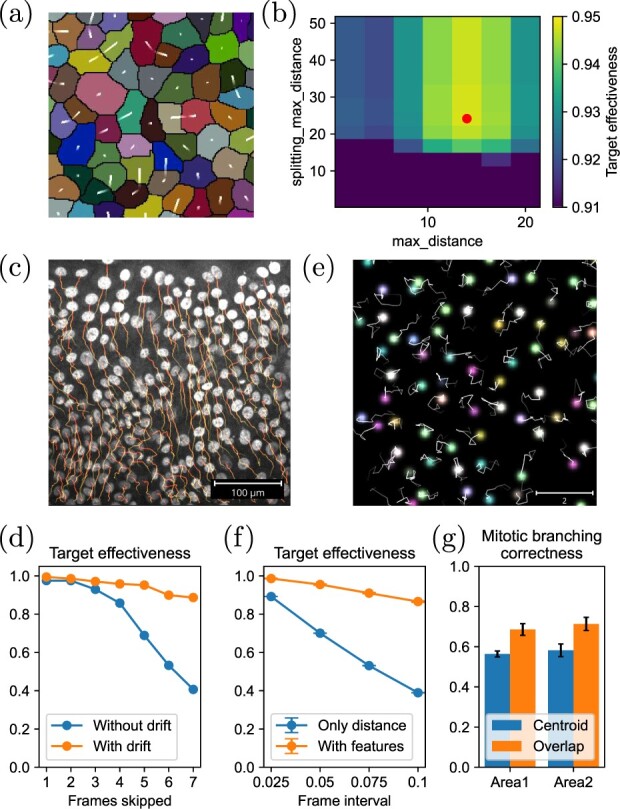
(**a**) An example snapshot for the mouse epidermis dataset. The white lines indicate the centroid displacement between frames. (**b**) TE as a function of max_distance and splitting_max_distance for the mouse epidermis dataset. The red point indicates the maxima. (**c**) An example snapshot for the cell migration dataset. (**d**) TE score for the cell migration dataset with skipped frames, with or without the drift term in the metric. (**e**) An example snapshot for the colored particles dataset. (**f**) TE score for the colored particles dataset with different frame intervals, with or without the feature difference term in the metric. The error bar indicates the standard deviation of five trials. (**g**) Mitotic branching correctness score for the mouse epidermis dataset, tracked with the centroid distances (centroid) or the overlap ratio (overlap). The error bar indicates the standard deviation of five trials

We first investigated the performance against various cost cut-off points in the simplest cases where the costs for connecting, gap closing and splitting are the squared Euclidean distance between the centroids. Specifically, we varied the maximum distance allowed for frame-to-frame particle association (max_distance) and splitting and gap-closing association (splitting_max_distance), which defines the cut-off for *l_ij_* and *s_αβ_* (*g_αβ_*), respectively, and investigated how the overall performance changes. In the mouse epidermis dataset (Fig. 2a), we performed grid search in the parameters max_distance and splitting_max_distance. We found that there exists a maxima in the TE around some finite length scale, suggesting that optimization is useful in performance improvement even for the cut-off parameters (Fig. 2b). We also found that the correlation of the tracking scores between mouse epidermis data from different regions are high upon changing of the parameters [*r *=* *0.96 (*r *=* *0.90) for TE (TP) using data with TE>0.75 (TP>0.75), respectively (Supplementary Fig. S1)], meaning that the optimized parameters are transferable within similar data.

### 3.2 Distance-only LAP tracker can achieve comparable performance to data-specific methods

We then benchmarked the tracking performance of the simple distance-only LAP tracker with the Yeast Image Toolkit dataset. Since the published benchmark results in Versari *et al.* (2017) do not include divisions, we tracked ground truth segmentation positions without splitting, with different cut-off points max_distance and gap_closing_max_distance (the cut-off point for *g_αβ_*). We then calculated the TE, the assignment F-score (tracking F-score), and the F-score for the assignments between the first and the last frames (long-term tracking F-score). We used the *Evaluation Platform* software (Versari *et al.*, 2017) to calculate the F-scores. Supplementary Figure S2 shows that this simple tracker achieves TE higher than 0.9 for all the datasets, and the F-scores are comparable to or higher than most published methods (Versari *et al.*, 2017), except the long-term tracking F-score for TestSet 3 and 4, which have frames with large cell displacements. Note that the previous methods track the cells after their segmentation pipeline, whereas we started with the ground-truth segmentation which can be advantageous. Nevertheless, this result suggests that the distance-based LAP tracker can generate tracks with accuracy comparable to data-specific tracking methods, as long as we start with sufficiently accurate segmentation.

We also performed a similar benchmark with the C2C12 dataset and found that the distance-only tracker yields the maximum TE of 0.998 when starting from ground-truth segmentation (Supplementary Fig. S3). This is higher than the score from the cutting-edge graph neural network-based tracking method; 0.976, which was obtained from the test including segmentation and in a larger dataset (Ben-Haim and Riklin-Raviv, 2022).

### 3.3 Tunable cost function improves tracking performance

We next investigated if variable cost functions help improve the tracking score for different datasets.

In Figure 2c, we show a snapshot of the cell migration dataset. Here, the cells are moving collectively toward the upper open region. Due to this drift, LAP-based tracking based solely on Euclidean distance fails with large frame intervals, as demonstrated in Figure 2d using datasets with skipped frames. This situation can be easily fixed by changing the cost function by adding a drift term to the Euclidean distance as

(3) $$l(x_{i},x_{j})={\left\|x_{i}-x_{j}+d\right\|}_{2}^{2}$$

with the drift parameter $d\in R^{2}$ and defining *g* and *s* analogously (Fig. 2d, Supplementary Fig. S4). We used 5% of the non-dividing and dividing connections to tune *d* as well as the cut-offs so that they optimize the true positive rate of the connections. The details are summarized in the Supplementary Material.

In real experimental data, particles may have features that help to identify species, such as the size, shape, and fluorescent intensities of genetic labels. In those cases, we can use those features in addition to the Euclidean distances to improve the performance. To illustrate this, we measured the tracking performance for simulated particles with eight species, characterized by different sets of feature values corresponding to RGB colors (Fig. 1e, see Section 2.1.5 for details). We then defined the cost function as

(4) $$l(\left\{x_{i},c_{i}\right\},\left\{x_{j},c_{j}\right\})={\left\|x_{i}-x_{j}\right\|}_{2}^{2}+w{\left\|c_{i}-c_{j}\right\|}_{2}^{2},$$

where $c_{i},c_{j}\in R^{3}$ are the feature vectors. We tuned the parameter *w* as well as the distance cut-off using the training data with 100 frames so that the tracking result maximizes the connection Jaccard index. We then measured the tracking scores for an independent dataset with 100 frames. As shown in Fig. 2f, with the features used in the metric, the target effectiveness with large frame interval remains above 0.8 while it drops to ∼0.4 when only Euclidean distance is in the metric (*w *=* *0), illustrating the performance improvement by including the particle features. We also observed an improvement in the other scores (Supplementary Fig. S5).

For segmented images, we can also use the overlap between segmented regions to calculate the cost (Chalfoun *et al.*, 2010; Ershov *et al.*, 2022; Padovani *et al.*, 2022). The flexible implementation allows us to integrate the overlap metric in addition to the distance in the LAP framework. We define *l* (with *g* and *s* analogously) as

(5) $$l(L_{i},L_{j})=-\text{log}\;\left(\frac{\frac{\left|L_{i}\cap L_{j}\right|}{\left|L_{j}\right|}+A}{1+A}\right)$$

which measures the overlap, where *L_i_* and *L_j_* are the set of pixel coordinates of the segmentation area for the particle *i* and *j* and *A* is a parameter. By comparing the tracking performance for the mouse epidermis dataset with the squared centroid Euclidean distance cases, we found that replacing the metric improves the mitotic branching correctness by ∼10% (Fig. 2g).

## 4 Conclusion

In this article, we showed how the LAP-based tracking pipeline with additional flexibility and optimizability can be useful in improving tracking performance in certain situations, and easily combined with visualization tools to conduct manual corrections. LapTrack, in large part, is complementary to TrackMate (Ershov *et al.*, 2022), which has a useful graphical user interface, support for including feature value differences, and its own optimization pipeline. Compared with TrackMate, LapTrack can take arbitrary inputs and cost functions and is flexible in its output, making it easier to connect with other upstream and downstream analysis pipelines. Trackpy (Allan *et al.*, 2021) provides a tracking routine based on the algorithm by Crocker and Grier (1996) in Python, as well as functions for particle detection, analysis and data input/output. One major difference is LapTrack’s ability to detect splitting and merging particles, which makes it more suitable for cell tracking. The tracking function in LapTrack is designed to help make accurate and validated tracks quickly and efficiently, with the hope to increase the amount of ground-truth data that can be used in training more sophisticated tracking methods.

With a sufficient amount of manually annotated ground-truth data, machine learning-based approaches will likely outperform the current parameter optimization strategy of simple affinity metrics. Due to its flexibility, our package can be easily combined with strategies such as one-to-one association affinity learning (Emami *et al.*, 2020; Li *et al.*, 2009), structured learning (Lou and Hamprecht, 2011), and the metric learning approach combined with graph neural networks (Weng *et al.*, 2020), serving as a reusable platform for implementation.

## Supplementary Material

btac799_Supplementary_Data

## Data Availability

The data underlying this article are available in GitHub at https://github.com/yfukai/laptrack and https://github.com/NoneqPhysLivingMatterLab/laptrack-optimisation, and archived in Zenodo at https://doi.org/10.5281/zenodo.5519537 and https://doi.org/10.5281/zenodo.7435087.
