# Supplementary material for: LapTrack: linear assignment particle tracking with tunable metrics
Source: Bioinformatics. 2022 Dec 10;39(1):btac799. doi: 10.1093/bioinformatics/btac799 (PMC9825786; doi:10.1093/bioinformatics/btac799)
Supplement: btac799_Supplementary_Data [file btac799_supplementary_data.zip › supplementary.pdf]

Supplementary Material for "LapTrack: Linear assignment particle tracking with tunable metrics"

Yohsuke T. Fukai [1,*] and Kyogo Kawaguchi [1,2,3]

[1] Nonequilibrium Physics of Living Matter RIKEN Hakubi Research Team, RIKEN Center for Biosystems Dynamics Research, Kobe, 650-0047, Japan.
[2] RIKEN Cluster for Pioneering Research, 2-2-3 Minatojima-minamimachi, Kobe, 650-0047, Japan.
[3] Universal Biology Institute, The University of Tokyo, Tokyo, 113-0033, Japan.

*To whom correspondence should be addressed.

Supplementary Text 1: LAP implementation

The tracking algorithm is formulated following Jaqaman *et al.* (2008).

Frame-to-frame LAP

Let the points in frame t be $x_i (i = 1 \dots M)$ and the points in frame $t+1$ be $y_i (i = 1 \dots N)$. Let us define the matrix $\mathrm{diag}^\infty(a)$ by

$$\mathrm{diag}^\infty(a)_{ij} := \begin{cases} a & (i=j) \\ \infty & (\text{otherwise}). \end{cases} \tag{S1}$$

We define the frame-to-frame cost matrix C^{ff} by

$$C^{\mathrm{ff}} = \begin{pmatrix} L & \mathrm{diag}^\infty(d) \\ \mathrm{diag}^\infty(b) & L' \end{pmatrix} \tag{S2}$$

where L and L' are $M \times N$ and $N \times M$ matrices, respectively, defined by

$$L_{ij} = \begin{cases} l_{ij} & (l_{ij} < \hat{l}) \\ \infty & (\text{otherwise}) \end{cases} \tag{S3}$$

and

$$L'_{ji} = \begin{cases} l_0 & (L_{ij} < \infty) \\ \infty & (\text{otherwise}) \end{cases} \tag{S4}$$

where $l_{ij} = l(x_i, y_j)$, $l_0 = \min(L_{ij}, b, d)$, and $\hat{l}$ is the cost cut-off (`track_cost_cutoff`).

We then solve the LAP by the LAPJVsp algorithm (Jonker and Volgenant, 1987) implemented as `min_weight_full_bipartite_matching` function in SciPy (Virtanen *et al.*, 2020) to minimise the overall cost

$$L_{\mathrm{ff}} = \sum_{i,j} C^{\mathrm{ff}}_{ij} A_{ij} \tag{S5}$$

where $\{A_{ij}\}$ is the assignment matrix satisfying

$$A_{ij} \in \{0, 1\} \tag{S6}$$

$$\sum_i A_{ij} = 1 \tag{S7}$$

$$\sum_j A_{ij} = 1. \tag{S8}$$

The points x_i, y_j are regarded connected when $A_{ij} = 1$.

Segment connecting LAP

Let the track segments generated in the frame-to-frame LAP be $z_\alpha (\alpha = 1 \dots J)$ and the first (last) frame and the coordinates of z_α be $t^{(s)}_\alpha$ ($t^{(e)}_\alpha$) and $z^{(s)}_\alpha$ ($z^{(e)}_\alpha$), respectively. Let the frames and the coordinates of the all middle points of the track segments be t_α and $w_\alpha (\alpha = 1 \dots K)$.

Similarly, the segment-connecting cost matrix C^{sc} is defined by

$$C^{\mathrm{sc}} = \begin{pmatrix} G & M & \mathrm{diag}^\infty(d) & \infty \\ S & \infty & \infty & \mathrm{diag}^\infty(d') \\ \mathrm{diag}^\infty(b) & \infty & \infty & S' \\ \infty & \mathrm{diag}^\infty(b') & M' & G' \end{pmatrix} \tag{S9}$$

where the entries are defined as follows.

G, M, and S are $J \times J$, $J \times K$ and $K \times J$ matrices, respectively, defined by

$$G_{\alpha\beta} = \begin{cases} g_{\alpha\beta} & (g_{\alpha\beta} < \hat{g} \text{ and } t^{(e)}_\alpha < t^{(s)}_\beta \le t^{(e)}_\alpha + \Delta t) \\ \infty & (\text{otherwise}), \end{cases} \tag{S10}$$

$$M_{\alpha\beta} = \begin{cases} m_{\alpha\beta} & (m_{\alpha\beta} < \hat{m} \text{ and } t_\beta = t^{(e)}_\alpha + 1) \\ \infty & (\text{otherwise}), \end{cases} \tag{S11}$$

$$S_{\alpha\beta} = \begin{cases} s_{\alpha\beta} & (s_{\alpha\beta} < \hat{s} \text{ and } t_\alpha = t^{(s)}_\beta - 1) \\ \infty & (\text{otherwise}), \end{cases} \tag{S12}$$

where $g_{\alpha\beta} = g(z^{(e)}_\alpha, z^{(s)}_\beta)$, $m_{\alpha\beta} = m(z^{(e)}_\alpha, w_\beta)$, $s_{\alpha\beta} = s(w_\alpha, z^{(s)}_\beta)$, Δt is the maximum allowed gap size (`gap_closing_max_frame_count`), and $\hat{g}$, $\hat{m}$, and $\hat{s}$ are the cost cut-offs (`gap_closing_cost_cutoff`, `merging_cost_cutoff`, and `splitting_cost_cutoff`).

G', M', and S' are defined by

$$G'_{\beta\alpha} = \begin{cases} l'_0 & (G_{\alpha\beta} < \infty) \\ \infty & (\text{otherwise}), \end{cases} \tag{S13}$$

$$M'_{\beta\alpha} = \begin{cases} l'_0 & (M_{\alpha\beta} < \infty) \\ \infty & (\text{otherwise}), \end{cases} \tag{S14}$$

© The Author 2022. Published by Oxford University Press. All rights reserved. For permissions, please e-mail: journals.permissions@oup.com

and

$$S'_{\beta\alpha} = \begin{cases} l'_0 & (S_{\alpha\beta} < \infty) \\ \infty & (\text{otherwise}), \end{cases} \tag{S15}$$

where $l'_0 = \min\left(G_{\alpha\beta}, M_{\alpha\beta}, S_{\alpha\beta}, b, d, b', d'\right)$.

The LAP is solved in the same way and the gap closing, splitting, and merging connections are added when the corresponding entry of the assignment matrix is 1.

Supplementary Text 2: Parameter optimization

We optimized the parameters using Ray Tune `https://www.ray.io/ray-tune` (Moritz *et al.*, 2018) with `BasicVariantGenerator` (random search) and `OptunaSearch` (Akiba *et al.*, 2019). Ten rounds of ten parallel parameter estimation were performed for each method, where the maximum allowed gap size was scanned from 0 to 1. The parameter ranges and initial values were set as follows.

Distance cut-offs and drift parameter d

Let $\mathcal{E}$ be the set of all given training connections with all the entry $(x, y) \in \mathcal{E}$ aligned so that x exists in an earlier frame than y does. The range of values and the initial value for the distance cut-off points were set as $[1.5 \times q^{50\%}, 1.5 \times q^{99.9\%}]$ and $1.5 \times q^{90\%}$, respectively, where $q^{a\%}$ is the a percentile of all connection distances $\left\{\|x - y\|_2 \,|\, (x, y) \in \mathcal{E}\right\}$. The cost cut-off points were then set as the square of those values. When optimizing the parameters in Eq. (4) and (5), the initial values of the distance cut-off points were set as those with the highest connection Jaccard index in the squared centroid Euclidean distance cases.

For the cell migration dataset, the range of values and the initial value for the parameter d_j $(j = 1, 2)$ was set as $[m_j - s_j, m_j + s_j]$ and m_j, respectively, where m_j and s_j are the mean and the standard deviation of the values $\{(y_j - x_j) |\, (x, y) \in \mathcal{E}\}$.

Feature weight w

The range of values and the initial value of the parameter w were set to $[0, 10]$ and 0, respectively.

Overlap cost function

The range of values and the initial value of the parameter A were set to $[0.01, 0.5]$ and 0.01, respectively. The cost cut-off point was fixed to $-\log(0.01)$. Distance cut-off points were estimated as previously, and costs are regarded as infinity for the points whose centroid distance is larger than the cut-off.

Supplementary Figures

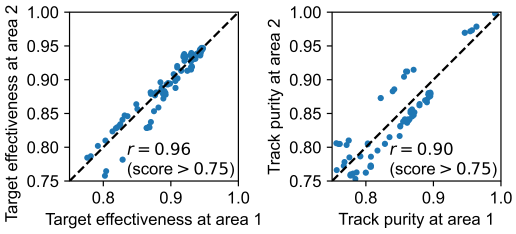

Fig. S1. The tracking scores for the mouse epidermis dataset at different regions with varied distance cut-off points [Fig. 2(b)]. The black broken lines indicate the equal scores. The Pearson correlation coefficient r for data with the score values > 0.75 is noted in the plot.

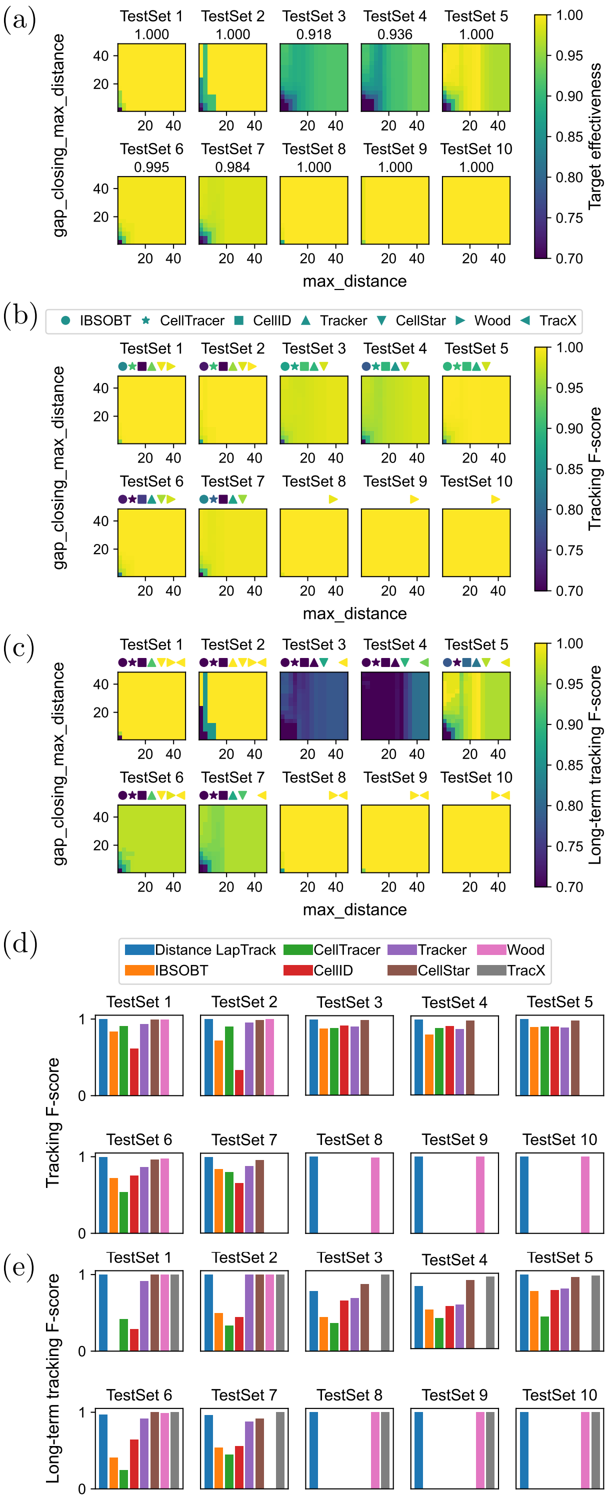

Fig. S2. The tracking scores for the Yeast Image Toolkit dataset with varied distance cut-off points. (a) The target effectiveness. The number above each plot is the maximum score. (b) The tracking F-score. The colors of the symbols at the top indicate the scores of the previously reported methods (Versari et al., 2017). (c) The long-term tracking F-score. The colors of the symbols at the top indicate the previous scores in Versari et al. (2017). (d) The best tracking F-score of the distance-only LAP tracker (leftmost), compared with the scores of the other methods. (e) The best long-term tracking F-score of the distance-only LAP tracker (leftmost), compared with the scores of the other methods.

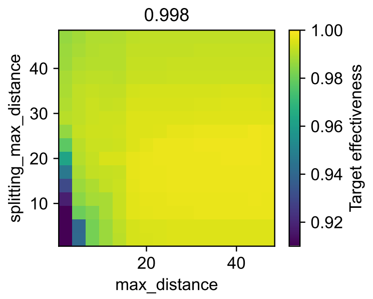

Fig. S3. The target effectiveness for the C2C12 dataset with varied distance cut-off points. The number at the top (0.998) is the maximum score.

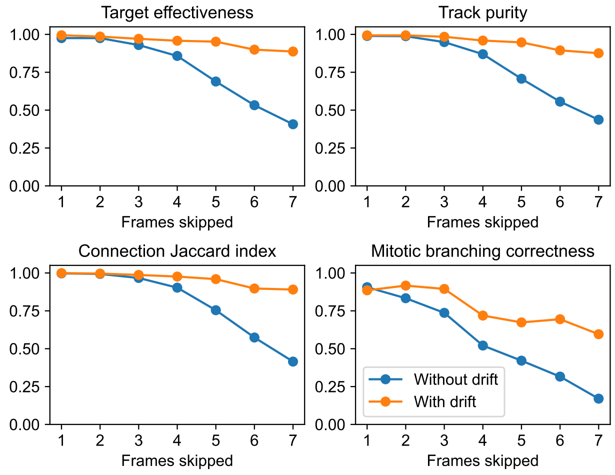

Fig. S4. All tracking scores for the cell migration dataset.

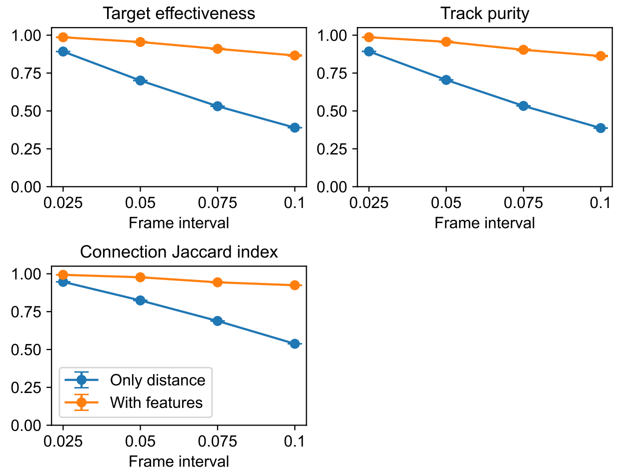

Fig. S5. All tracking scores for the coloured particles dataset.

References

Akiba, T., Sano, S., Yanase, T., Ohta, T., and Koyama, M. (2019). Optuna: A Next-generation Hyperparameter Optimization Framework. In *Proceedings of the 25th ACM SIGKDD International Conference on Knowledge Discovery & Data Mining*, KDD '19, pages 2623–2631, New York, NY, USA. Association for Computing Machinery, 10.1145/3292500.3330701.

Jaqaman, K., Loerke, D., Mettlen, M., Kuwata, H., Grinstein, S., Schmid, S. L., and Danuser, G. (2008). Robust single-particle tracking in live-cell time-lapse sequences. *Nature Methods*, **5**(8), 695–702. 10.1038/nmeth.1237.

Jonker, R. and Volgenant, A. (1987). A shortest augmenting path algorithm for dense and sparse linear assignment problems. *Computing*, **38**(4), 325–340. 10.1007/BF02278710.

Moritz, P., Nishihara, R., Wang, S., Tumanov, A., Liaw, R., Liang, E., Elibol, M., Yang, Z., Paul, W., Jordan, M. I., and Stoica, I. (2018). Ray: A distributed framework for emerging AI applications. In *13th USENIX Symposium on Operating Systems Design and Implementation (OSDI 18)*, pages 561–577, Carlsbad, CA. USENIX Association.

Versari, C., Stoma, S., Batmanov, K., Llamosi, A., Mroz, F., Kaczmarek, A., Deyell, M., Lhoussaine, C., Hersen, P., and Batt, G. (2017). Long-term tracking of budding yeast cells in brightfield microscopy: CellStar and the Evaluation Platform. *Journal of The Royal Society Interface*, **14**(127), 20160705. 10.1098/rsif.2016.0705.

Virtanen, P., Gommers, R., Oliphant, T. E., Haberland, M., Reddy, T., Cournapeau, D., Burovski, E., Peterson, P., Weckesser, W., Bright, J., van der Walt, S. J., Brett, M., Wilson, J., Millman, K. J., Mayorov, N., Nelson, A. R. J., Jones, E., Kern, R., Larson, E., Carey, C. J., Polat, İ., Feng, Y., Moore, E. W., VanderPlas, J., Laxalde, D., Perktold, J., Cimrman, R., Henriksen, I., Quintero, E. A., Harris, C. R., Archibald, A. M., Ribeiro, A. H., Pedregosa, F., van Mulbregt, P., and SciPy 1.0 Contributors (2020). SciPy 1.0: Fundamental Algorithms for Scientific Computing in Python. *Nature Methods*, **17**, 261–272. 10.1038/s41592-019-0686-2.
